# Supplementary material for: Identification of photoperiod-induced specific miRNAs in the adrenal glands of Sunite sheep (Ovis aries)
Source: Front Vet Sci. 2022 Jul 22;9:888207. doi: 10.3389/fvets.2022.888207 (PMC9354845; doi:10.3389/fvets.2022.888207)
Supplement: Supplementary file 1 [file Data_Sheet_1.ZIP › Supplementary file/miRDeep2 file.zip!/miRDeep2 file/Novel_Structure/SPLP42A3/Novel_384.pdf]

Provisional ID	:	NC_019466.2_18216
Score total	:	13.4
Score for star read(s)	:	3.9
Score for read counts	:	7.3
Score for mfe	:	0.6
Score for randfold	:	1.6
Score for cons. seed	:	
Total read count	:	26
Mature read count	:	23
Loop read count	:	0
Star read count	:	3

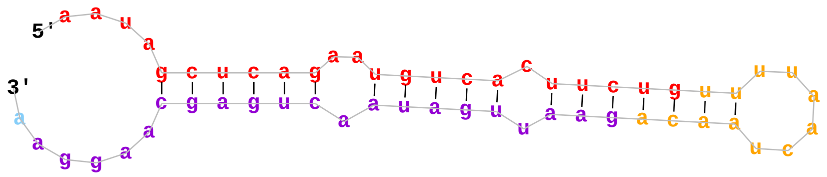

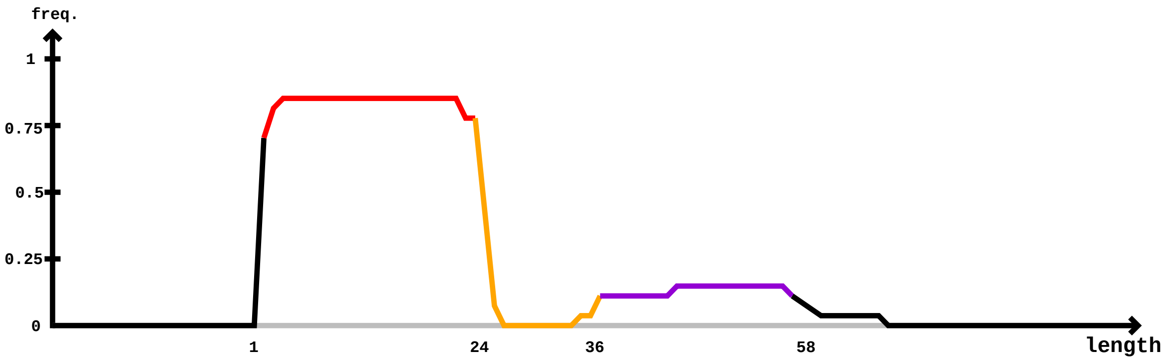

Mature Star

```
5'- uuuguaauucuuuacuguggaauagcucagaaugucacuucuguuuuaacuaacagaauugauaacugagcaaggaaacguaauuuggauuauaaaauucuugcuuuaaua   -3'    obs
    uuuguaauucuuuacuguggaauagcucagaaugucacuucuguuuuaacuaacagaauugauaacugagcaaggaaacguaauuuggauuauaaaauucuugcuuuaaua          exp
    ((((((((((.((((.((......((((((..(((((.(((((((......))))))).))))).))))))......))))))...)))))))))).............    reads    mm    sample
    ....................aauagcucagaaugucacuuc......................................................................    2        0     seq
    ....................aauagcucagaaugucacuucug....................................................................    17       0     seq
    .....................auagcucagaaugucacuucug....................................................................    2        0     seq
    .....................auagcucagaaugucacuucuguu..................................................................    1        0     seq
    ......................uagcucagaaugucacuucuguu..................................................................    1        0     seq
    .....................................................cagaauugauaacugagcaagg....................................    1        0     seq
    .......................................................gaauugauaacugagcaagga...................................    2        0     seq
    ...............................................................aacugagcaaggaaacguaauu..........................    1        0     seq
```
